# The Changing Landscape: High-Level Influences on Eye Movement Guidance in Scenes

**DOI:** 10.3390/vision3030033

**Published:** 2019-06-28

**Authors:** Carrick C. Williams, Monica S. Castelhano

**Affiliations:** 1 Department of Psychology, California State University San Marcos, San Marcos, CA 92069, USA; 2 Department of Psychology, Queen’s University, Kingston, ON K7L 3N6, Canada

**Keywords:** eye movements, scenes, attention

## Abstract

The use of eye movements to explore scene processing has exploded over the last decade. Eye movements provide distinct advantages when examining scene processing because they are both fast and spatially measurable. By using eye movements, researchers have investigated many questions about scene processing. Our review will focus on research performed in the last decade examining: (1) attention and eye movements; (2) where you look; (3) influence of task; (4) memory and scene representations; and (5) dynamic scenes and eye movements. Although typically addressed as separate issues, we argue that these distinctions are now holding back research progress. Instead, it is time to examine the intersections of these seemingly separate influences and examine the intersectionality of how these influences interact to more completely understand what eye movements can tell us about scene processing.

## 1. Introduction

Visual scenes are ubiquitous but complex concepts. Scenes are typically defined as any view of the natural world [1,2,3,4,5,6]. However, we will adopt a narrower definition often used by researchers (e.g., [3]), where a scene is defined as a human-scaled view of an environment that is made up of space-defining surfaces and larger elements [7], containing a number of smaller objects arranged in specific spatial locations [8,9,10], and forming a coherent semantic concept [1,2,3]. Each component of this definition can contribute individually to the understanding of the visual world [1].

Breaking down the definition, the surfaces and larger elements that define the shape of the space of an environment have been shown to be crucial for the initial perceptual processing of scenes (i.e., spatial envelope [11,12,13]). This space, as defined by these larger elements, is the basic building block of the scene (e.g., floors, walls, and ceiling for indoor scenes). The shape and position of these elements have been linked to processes involved in navigation and visual search, and we will explore this in more detail below.

The arrangement of the smaller elements of a scene are relative to the space and position of larger space-defining elements. In addition, the arrangement follows the rules of physical objects (e.g., gravity), and this arrangement can affect both search and identification processing of individual elements [14,15]. Further, the position of these elements relative to the observer may have differing effects on processing [16,17].

The third component of the definition, semantic coherence, introduces the importance of prior knowledge. For example, knowing that the scene is a kitchen provides information that affects how the scene is represented based on the likelihood of objects present, functions performed in that space, and the likely spatial arrangement of certain elements [14,18,19].

The final aspect of the definition is that scenes are human-scaled environments. Considering that this is the way our real-world environment is normally viewed, it is reasonable to think that these views would be most familiar, easiest to process, and most relevant for considering different factors having an effect on processing. Although researchers can examine other perspectives, such as those that are either too small (e.g., the world from the view of a cat) or too large (e.g., a satellite image of a city), there are visual and structural properties that are available at the human scale that would not be available with other views.

The above definition points to the complexity of trying to encompass all aspects of real-world scenes in a single definition. However, we do acknowledge that this definition is strongly linked with the stimuli used to depict scenes in labs (i.e., photographs, line drawings, computer generated images) and does not reflect the changing scales and embedded nature of scene perception that occurs when we are perceiving, processing, and navigating a real-world environment. We will come back to this limitation later and discuss potential ways that future research can address it. Although the issues are complex, how we view and interpret scenes is important in our understanding of human visual behavior. The question then is how to assess the visual evaluation of scenes. One can approach the problem multiple ways; we will focus on the use of eye movements to examine how scenes are processed and understood. Rayner and Pollatsek [5] reviewed the extant literature on eye movements and scene perception in the early 1990s. Critically, although most of their research focuses on reading, Rayner and Pollatsek laid out some basic questions that eye movements could answer regarding scene processing. They point to the tension (that still exists) between the processing of varied and complex visual information from scenes and the effect of task on what type of information is acquired and processed. Their review covered what was known at that time about scenes and eye movements, but in the last 30 years, the study of eye movements and scene perception has exploded. Researchers from around the globe have since explored the effects of attention on understanding and remembering scenes as well as the effects of scene knowledge and memory on the deployment of attention.

However, to gain traction on the complex processes involved in scene processing, researchers have made simplifying assumptions about scenes. For instance, scene context is known to have a strong influence on behavior, but the exact nature of that influence is rarely specified, even though it could arise from the various sources as described in the definition above. The field as a whole has begun to recognize that these simplifying assumptions may limit the explanatory power of theories and as such, limit our knowledge of scene processing to a relatively narrow case of visual processing.

The present review will examine the current state of research on scene processing and its development over the last 10 years with a focus on eye movements. Although our review will necessarily be selective, we hope to highlight not only how some of the simplifying assumptions made in the past may have run their course but also how, to move forward, research in eye movements and scene perception will need to embrace the intersectionality of how different influences and sources interact.

In this review, we examine the processing of real-world scene information through eye movements to explore the timeline of processes in more fine-grained units and explore what processes are critical in scene viewing. The eye movement record of a person viewing a scene allows researchers to examine: (1) attention and eye movements; (2) where you look; (3) influence of task; (4) memory and scene representations; and (5) dynamic scenes and eye movements. We address each point in turn.

### Why Use Eye Movements to Study Scenes?

Although eye movements provide an excellent record of the location and timing of where the eyes are pointed, this record would be meaningless unless we could tie the eye movement record to mental processes. Early demonstrations showed that eye movements reflected different ways information was processed [20,21], and researchers have consistently since found that eye movements are tied to cognitive processing and attention [22,23,24]. When the eyes move, attention precedes the eyes to the intended location and remains at that location for some time before moving to another location (potentially the location of the next saccadic target). Although not absolute (attention can and does move to locations that the eyes do not travel to), the location of a fixation is known to be an attended location. By taking advantage of the various fixated locations, researchers can get a better understanding of why those spatial locations were attended and as well as the order of those selections. Based on the definition of a scene provided above, eye movements can provide many advantages that other measurements cannot:

1. **Eye movements are natural.** Because of the structure of the eye, people naturally move their eyes to point the location of the highest acuity in the retina (i.e., the fovea, ~2° of visual angle) at what they wish to “look” at (see Kowler [25] for a more detailed description of the visual field). To compensate for this limited area of high acuity, people rotate the eyes to focus light from different physical locations onto the fovea. Importantly, in contrast to cognitive tasks that require the experimenter to train the participant in how to respond correctly, researchers do not teach participants how to move their eyes. In fact, it takes effort and monitoring by the researcher if the goal is to have participants not move their eyes. Most people are unaware that the eyes move a number of times per second (~3 eye movements per second [26]). Although not completely implicit, the relative ease of eye movements and their relative “invisibility” makes them an ideal tool for non-invasively observing behavior. In addition, the measurement of eye movements has become relatively easier in the last two decades with the costs of eye trackers falling and the ease of use of these devises increasing. Overall, eye movements provide a low-cost way to unobtrusively observe natural behavior.
2. **Eye movements are fast.** Eye movements and fixations operate on a time scale that allows researchers to have greater precision in their measurements. Saccadic eye movements generally take less than 50 ms (frequently much less) to rotate the eyes from pointing at one part of the visual world to pointing at another part of the visual world. Once the eyes have rotated to point to the new location, they pause or fixate for a brief amount of time (e.g., 100–400 ms). While the eye is in motion, visual processing from the eyes is limited through saccadic suppression [26], so of cognitive interest is when the eye is relatively still (such as during a fixation) and visual information is acquired. Borrowing from reading research, scene processing has been measured using different fixation measures based on duration, number/count, and location. However, aggregate fixation measures that define processing across different temporal windows have proven to be especially useful. For instance, gaze duration (the sum of the fixation durations on a region of interest from the first fixation in the region to when the eyes leave that region) can give an indication of the time to initially process and recognize an object. Subsequent fixations (second gaze duration or total time) would indicate that additional information gathering was needed or that a checking/confirming process was necessary.
3. **Eye movements operate across a spatial dimension.** Unlike temporal measures that are inherently unidimensional (e.g., reaction time), measuring where the eyes are directed allows researchers to determine areas of a stimulus that the participant is currently prioritizing. The spatially distributed information of eye movements allows researchers to have a direct measure of prioritization of information available to the observer, examine commonalities in prioritization across individuals, and allow for other interesting spatially aggregate measures. For instance, the proportion of the image fixated and the scan path length can each indicate the extent of exploratory vs. focused behavior. Some tasks encourage greater exploration of the scene (e.g., memorization), whereas others constrain that exploration (e.g., visual search). Further, scan path allows for a direct measure of efficiency of the eye movements as it can be used to create a ratio of the distance taken to reach a critical region to the shortest distance possible. Thus, with all 360° of possible prioritization for the next fixation, the spatial dimension allows for a rich set of measures that reflect different types of processing.
4. **Eye movements operate across a temporal dimension**. Because the eyes have to move from one location to the next in a serial manner, eye movement data also provide a temporal record of processing in addition to the spatial record. This information allows researchers to identify the order that scene features are processed, potentially indicating their relative importance to the task. In addition, fixations typically last only a few hundred milliseconds, which is much shorter than many complex tasks (e.g., search) take to complete. The serial fixation record can be examined to determine, at a more fine-grained time scale, the processing that was occurring at each point in the trial rather than on a global scale (i.e., reaction time).

All of these features make eye movements an especially useful tool for studying the processing that occurs in scenes. The ability to create a record of the spatial locations visited and the timing of those visits with a measurement that is practically implicit to the participant allows researchers to probe scene processing in ways that other methods do not possess. As mentioned above, the fact that fixation location is linked to attentional processing is critical to the use of eye movements as a cognitive measure. The following section examines research into this connection with regard to scene processing.

## 2. Attention and Eye Movements

Researchers have demonstrated that there is a tight link between eye movements and attention [27,28,29,30]. From this, researchers have also established that attention is tightly linked with the planning and execution of an eye movement to a new location in the environment. For instance, in a seminal study, Hoffman and Subramaniam [23] demonstrated that attending to one location while saccading to another did not improve performance at the attended location, but instead improvement was seen at the location of the saccade landing point. In recent years, researchers have highlighted exceptions to this assumed link between attention and saccade targets [27,28,29]. For instance, Golomb et al. [31,32,33] revealed facilitation effects at both retinotopic and spatiotopic coordinates. They argue that a lingering retinotopic trace persists after the eyes have moved, and it persists for a small window of time with the updated spatiotopically relevant area. This research highlights not only the connection between attention and eye movements but also the temporal shifts of processing within a fixation as the current fixation shifts to a new location.

Interestingly, the demonstration of the connection between eye movements and attention also presents an interesting conundrum for the interpretation of fixation durations. As stated above, the decision of where to move the eye is inherently a part of measurements that reflect when to move the eyes. Thus, the link between eye movement and attentional processes are not a straightforward causal relationship, as many early studies posited [34]. These more recent studies do not dispute the link between attention and eye movements, but rather highlight how information processing at different positions relative to the current eye position are updated over time and introduce a more fractured view of the role of attention relative to eye movements.

Although it is true that stimulus properties are often researched as the main driving force influencing where we look, there also seems to be a shift in the understanding of how stimulus properties drive eye movement location. Other influences on the direction of attention and eye movements have recently come to the fore. For instance, internal tendencies for how information is acquired seems to have an independent effect on eye movement guidance [35,36,37,38]. As an example, there is the central bias effect [36], where the eyes have a bias to look to the center of the image. Although initial eye movements seem to be positioned centrally, this effect dissipates with time [35,39], but other factors influence it as well. For instance, Bindeman [35] found that both the center of the scene and the screen itself play a role in the bias, as only early eye movements were directed to the center. Furthermore, Rothkegel et al. [39] had participants initially fixate on a scene to one side and found that the initial fixation voluntarily made by participants was not affected by the scene context because it was independent of the location of the most informative regions. Inherent in the interpretation of the central bias is the notion (either explicitly or implicitly) that the attentional window starts wide and then focuses on scene details. This default mode of processing is ubiquitous regardless of stimuli details. We assume attention and eye movements interact in the selection, planning, and execution of a new voluntary eye movement. It is how we select the information to be scrutinized that we now turn to.

## 3. Where You Look

By far, the largest number of research studies on eye movements and scenes have examined the question of where we look. The driving force behind this question is of course that certain types of information are prioritized for further inspection or that certain characteristics of the environment tend to capture attention (or draw attention) to them. Although there is still some focus on how stimulus properties capture attention, more recent studies have introduced a number of new ways of thinking about and categorizing the type of visual properties that receive further scrutiny through eye movement planning. We examine these different influences in turn, beginning with the omnipresent properties of the stimulus itself, and then exploring other factors.

### 3.1. Influence of Stimulus Properties

Traditionally, researchers have examined basic and higher-order image features and how they drive the eyes in a bottom-up manner. Computer vision has been incredibly influential in how researchers theorize about how eye movements are guided, most predominantly through computational models of saliency [40,41]. Visual saliency and saliency maps try to define areas that “stand out” from the background as potential points of interest. When looking at images, the eyes rarely go to large homogenous areas such as a blue sky or a blank wall [20,21,42]. Saliency calculations attempt to find the areas of the image based on the low-level features that can be extracted from the image itself. Saliency maps highlight the coordinates of the points that stand out and allow for a ranking of importance within the image. Low-level features such as color, orientation, and intensity [40,43], as well as second-order features such as intersections and edges [44,45] have been found to affect eye movement planning. Many researchers have explored the combined and separate contributions of low-level features to eye movement guidance (e.g., color [46]), but there has been movement away from a purely bottom-up approach.

Although saliency has inspired a number of theoretical models and research studies, over the past decade, the limits of saliency as an explanatory tool has become more pronounced [47,48,49]. First, inherent in models of saliency is the notion that information selection is passive and based solely on the properties of the image regardless of the individual’s intent. However, across many studies, it should be noted individuals are actively seeking visual input, regardless of task [49,50]. Second, there is an overall movement away from classifying influences as either purely top-down or bottom-up [49,51]. Instead, researchers have begun to examine different sources of information (e.g., immediate history with a task) and how those sources are combined and interact. For instance, many recent models are finding ways to incorporate higher-level information such as meaning and objects into how scene information is selected. There are many computational models that have since been proposed to better represent higher-order information, but review of that work is beyond the scope of the current paper. However, we will consider the different approaches for considering higher-order information and its influence on eye movement guidance.

### 3.2. Meaning or Object as the Unit of Selection

Rather than treating information as either purely top-down or bottom-up sources, researchers are finding that the combination of these factors best explain eye movement planning. One method has been to identify objects (rather than low-level features) as the unit of selection for eye movement planning [52,53,54,55]. This stems from early demonstrations that observers tend to prefer to focus on objects rather than background elements [20,21]. In these cases, objects are defined as meaningful entities that are visually distinct from the background. For instance, Stoll et al. [55] found that the preferred landing position on an object was centered on it in relation to the object’s boundaries. Unlike the low-level features that posit that local edges (changes in contrast) may attract attention, an object-centered approach conveys that the center of mass within those edges are the targets of eye movements (e.g., [56]). Indeed, Pereira and Castelhano [8] found that fixations were directed at groups of objects within a larger scene context during search, and they concluded that object content provided specific information about where to aim fixations.

The issue with using objects in calculating where to attend is the fact that it is often difficult to define what an object is from the image itself. The complexity of figure-ground separation is only magnified with multiple objects at multiple depths in scenes. Without defining objects, researchers have sought to update saliency models with other higher-level features. For instance, proto-objects can be used as the unit of selection [57,58,59,60,61]. Although the definitions vary across studies, there is a general consensus that proto-objects are defined as fragments of a feature-similar visual space or are pre-attentive structures with limited spatial and temporal coherence. These signals of objects (rather than relying on the separation of whole object representations) serve to circumvent the question of how you know the identity of what is there before you know that something is there (e.g., [62]). The proto-object approach also circumvents the distinction between purely bottom-up, low-level features and top-down priorities. For instance, Wischnewski et al. [60] argue that having proto-objects as the unit of incoming information allows for models to incorporate different aspects of the visual scenes that may not otherwise be possible (such as temporal-spatial changes over time). Thus, proto-objects allow for further integration of different sources of information without having to define individual objects per se.

Other methods have been to establish the ground-truth of informativeness or meaningfulness of scene regions [63,64]. For instance, Henderson and colleagues had a separate group of participants rate the meaningfulness of small regions of the image. Using these ratings, the allocation of eye movements to different regions of the scenes were predicted from their level of meaningfulness as derived from these rating studies. The concept of meaning in this case is somewhat related to the proto-object properties mentioned above, in that it allows for object parts or high-level features to be the unit of analysis. In addition, the combination of presenting participants with isolated regions of the scene and using their ability to interpret that information (to varying degrees) results in a combination of low-level features traditionally used in saliency map, with a top-down, high-level interpretation of those features.

### 3.3. Semantic Integrity within the Larger Scene Context

The influence of overall scene semantics on selection is typically examined by contrasting eye movements to semantically congruent and semantically incongruent objects within a scene context. Any differences in fixating congruent and incongruent objects would be the result of the scene semantics because incongruency, especially, is defined by the object-scene relationship. Although there are multiple ways in which semantic incongruency could influence eye movements, researchers have concentrated on two main questions: whether inconsistent objects attract attention to themselves from a distance and how inconsistent objects affect processing, once attended. We will examine each question in turn.

First examined by Loftus and Mackworth [65], the question of whether semantically inconsistent objects automatically attract attention has been studied for decades. Despite being intuitively appealing, subsequent studies produced mixed results [66,67,68,69], and this inconsistency has continued in more recent studies [14,70,71,72,73]. For instance, Võ and Henderson [71] found that objects that were inconsistent with the scene context (e.g., a computer printer on a stove in the kitchen) did not attract initial fixations, suggesting that participants were not immediately drawn to these objects. On the other hand, Lapointe and Milliken [73] found that there was a tendency for inconsistent objects to be detected more quickly during a change detection task. The difference in patterns of results illustrate important interactions between stimulus properties (e.g., the size of the critical object) and task (e.g., visual search vs. change detection), highlighting another instance where different types of scene properties interact.

The mixed results seem to be associated with object size. For instance, it is unclear across studies whether the object’s identity can be extracted from parafoveal or peripheral information. To the extent that object size is constrained by the limited availability of information peripherally, a decrease in that object’s ability to draw attention is seen [74,75,76]. This question links to the question of when, during the execution of an eye movement, object identity is extracted. Is it parafoveally just prior to fixation or only once the details have been directly fixated and processed? We will examine this question in more detail below.

With regards to the influence of scene semantics, is the question of how semantic inconsistencies affect processing once objects are fixated. This question is less controversial than the first in that it has been well established that inconsistent or incongruent objects do lead to longer scrutiny and overall longer processing times [66,67,77]. More recent studies have demonstrated similar patterns of results [14,70,78], although there have been some exceptions [73,79]. We can also examine the converse effects of scene semantics and examine how consistent semantic information can positively affect eye movement guidance [6,67,80,81]. One technique to examine the influence of scene information on eye movement guidance is the Flash Preview-Moving Window (FPMW) paradigm [80]. The FPMW paradigm has participants briefly view a scene image preview (250 ms, which is too brief to execute an eye movement). Following the brief scene preview, a target label is presented that the participant needs to search for in a scene. The search scene is then presented, but the participant’s view of the scene is limited to a ~4° radius window centered on current gaze position. Because the window is locked to gaze, the participant views the scene as if viewing it through a paper tube. In this way, rather than relying on the immediately available visual information extracted from the periphery, planning of eye movements outside the window would require observers to rely on the representation of the scene from that initial preview. By manipulating the relationships of the preview to the search scene, researchers have explored several aspects of how scene representations affect eye movement guidance. For instance, researchers have shown that the scene semantic category did little to improve search performance [70,80], that specific details about the scene seems to be important [8,14,80], details about the target help [18,70], and that extraction of useful information occurs quite quickly [82]. More recently, researchers have also examined the effects of domain expertise and interestingly found that when radiologists viewed chest x-rays, the previews provided much smaller benefit than would be expected based on search in scenes [83].

As was suggested by previous research, studies in the past decade have found that consistent objects lead to more efficient search performance [8,14,70,84,85,86]. Researchers posit that the semantic relatedness of the object not only to the scene context, but also to other objects in the scenes, led to faster search. For instance, Hwang et al. [84] used annotated photo images (from LabelMe Database [87]) to examine the contribution of semantically related objects to the guidance of eye movements. They found that there was a tendency for the next fixation to be programmed to an object that was semantically similar to the currently fixated object. Further, Mack and Eckstein [86] found that when semantically related objects were placed in close proximity, search was much faster. In a recent development, Võ, and colleagues [9] proposed a key role for certain larger objects (anchor objects) that are associated with other objects (e.g., stove and a pot). They demonstrated that the presence of anchor object led to more effective guidance during search. Because anchor objects are typically large scene elements, they fit with the earlier studies as they can be identified in the parafovea or periphery providing semantic guidance within the scene [84,88,89]. In addition, this information can also provide spatial information that could aid gaze guidance, which we will discuss next.

### 3.4. Influences of Spatial Associations

Scene shape has been closely linked to scene categories [7,10,11,12,13,90,91] and has been used to explain how eye movements are guided during visual search [6,8,14,81,92,93]. That overall scene structure has an influence on search and object processing has been known for some time [4,80,81,94,95]. For instance, Castelhano and Heaven’s [14] study included a factorial manipulation of scene semantics and spatial positioning of target objects. Rather than finding that spatial consistency was only useful within semantically consistent scenes, they found that both semantic and spatial information influence search independently and additively.

Researchers examining when objects are out of place in a scene (i.e., in a spatially inconsistent location), have found that visual search performance is slowed [14,15,18,72,96]. For instance, Hillstrom et al. [96] found that when target objects’ location switched from a spatially consistent location to an improbable (mug on floor) or an impossible (mug in the air) location between the preview and search scene, performance significantly worsened. Furthermore, Castelhano and Witherspoon [18] found a strong link between the target object’s function and its spatial location in the larger scene context. They found that when the functions of novel objects were learned, participants were able to locate them much more quickly than when only the visual features of the target object were known. Further research has shown the link between action, function, and spatial organization is thought to be strongly linked in scene representations [19].

More recently, Castelhano et al. [17,97,98] have argued for the importance of scene surfaces in guiding attention during visual search. The Surface Guidance Framework posits that attention is directed to surfaces in the scene most associated with the target object. For example, (1) upper (e.g., ceiling, upper walls), (2) middle (e.g., countertops, tabletops, desktops, stovetops), and (3) lower regions (e.g., floor, lower walls), are associated with specific objects: (1) upper (e.g., painting, wall clock), (2) middle (e.g., blender, book), and (3) lower (e.g., garbage bin, shoes). By dividing the scene into relevant and irrelevant surfaces, we can define target-relevant and target-irrelevant regions for any scene-object combination. This, in turn, allows for the examination of how previous knowledge about the scene context and its association affects processing across regions. For instance, Pereira and Castelhano [97] found that suddenly onset objects were more likely to capture attention when they appeared upon a target-relevant than a target-irrelevant surface. Thus, how attention is deployed is closely tied to the scene structure, where surfaces can act as a larger object-based region across which attention is allocated [99,100].

Interestingly, researchers have also begun to inquire about differences in processing across the spatial depth of a scene. For instance, recent studies have shown qualitatively different processing of spaces closer to the observer [16,17,101,102,103]. For instance, Castelhano and Fernandes [17] found that foreground information (from the center of the total scene depth to the position of the observer within the scene) had a great initial influence on initial scene perception than background information. Furthermore, Bonner and Epstein [101] have found that activity in the occipital place area (OPA) was linked to perceiving potential paths for movement in immediate surroundings. In addition, Josephs and Konkle [16] have found that the spaces that are reachable are represented qualitatively differently than objects and larger scene spaces. Given the qualitative differences in processing across depth, it stands to reason that information closer in depth may have different utility than information farther away and thus, may differently affect eye movement guidance and visual search in a scene. Indeed, in a recent study, Man and Castelhano [103] found a consistent effect of scene depth, where targets placed closer in space (in the foreground of the scene) were found faster and with fewer fixations than those placed in the background, regardless of semantic association and regardless of target size. Thus, across studies information is processed differently across scene depth.

Across the different influences on how eye movements are guided, one commonality is in how these influences are assessed. Much of the research on how high-level information is prioritized or captures attention is attained through tasks such as visual search, change detection, or free viewing. To some extent, the commonalities across tasks point back to the notion discussed by Rayner and Pollatsek [5] that when trying to understand scene processing, task may be irrelevant as the complex nature of the scene processing is required regardless of how the observer is processing that information. However, we also know from a number of studies that task plays a crucial role in how information is processed. We turn to the question of the influence of task next.

## 4. Effects of Task

Although the contributions of the elements discussed above have formed a large part of the research on eye movements and scenes in the last decade, the importance of task and task-relevance of scene information has become more apparent. Buswell [20] and Yarbus [21] both described the importance of the task that the observer is performing. Rayner and Pollatsek [5] raised the issue of task in scene viewing and identified its many challenges. In contrast to reading, scene perception is not simply one task; search, memorization, or “free viewing” can all be tasks performed on scenes. Each task can alter the eye movement pattern dramatically because different aspects of the scene are relevant to each task. One of the most famous of these demonstrations was performed by Yarbus [21]. In his study, Yarbus examined the eye movements of a participant when looking at a single stimulus, the painting “An Unexpected Visitor,” with different tasks. Yarbus found that the locations of fixations varied with the task (see also [104]). Castelhano et al. [50] replicated the finding that the locations of fixations varied with the viewing task, but they also found that some measures, like fixation durations, were task invariant. The finding that the task can dramatically alter what is viewed indicates that it can interact with other aspects of scene processing.

The question of the task is critical when interpreting eye movements on scenes. Across the scene processing literature, many types of tasks have been employed. For instance, to encourage participants to scan over the spatial extent of the entire scene, tasks that direct the participant minimally are typically used, such as free viewing [20,105,106], memorization [50,107], or aesthetic judgments [108,109]. These less focused tasks encourage participants to scan a large proportion of the scene as participants are uncertain of the importance of any particular detail. Buswell [20] demonstrated that when participants were instructed to “look at the pictures in their normal manner” (p. 136) fixations were widespread. However, this breadth is not of uniform density and tends to be focused on parts of the scenes containing meaningful objects [42,50,63,110]. In contrast to less focused tasks, tasks that involve focused processing, such as search and change detection, require participants to be more directed in their viewing in line with a specific goal. Buswell [20] demonstrated that when participants searched for a person in a window in the same scene that they had freely viewed previously, the fixations were concentrated on possible locations where the target could be. In general, the extent of the eye movements executed under search instructions tend to be more focused on possible areas of the scene that the object can occur [6]. Because search tasks necessitate that some aspects are relevant and some are not, they allow researchers to manipulate the relative importance of physical features or meaning through the specification of the search target. For example, Peacock et al. [64] had participants look for bright patches in a scene (a physical feature), but they found that even though the search task did not encourage processing of the meaning of the objects in the scene, meaningfulness continued to affect where people looked in the scene. In addition, when the search target is absent, participants will search for an extended period of time, examining progressively more of the image and allowing for a more direct comparison of the scene processing between search and memorization (e.g., [111]).

Consistent with the theme of the current review, it is impossible to divorce viewing task from the type of information obtained while viewing a scene. The viewer’s task acts as a filter that highlights aspects of the scene that are consistent with the current goal. In some instances, the goal is relatively undefined like free viewing, which leads to fixation patterns that vary widely. On the other hand, focused goals like search lead to a more constrained viewing pattern. Regardless of the specific pattern, it is impossible to examine eye movements in scene processing without considering the task.

## 5. Influence of Scene Representations in Memory

As stated above, fixations on an image provide a record of the objects and areas that are attended to in the image. Because of this connection, the fixation record provides a reliable indication of aspects of a scene that could be encoded into memory [112]. In addition, the serial nature of eye movements allows researchers to examine the order of encoding information into memory. Given these two facets, the influence of eye movements on memory could actually be bi-directional: in one direction, a previously encountered scene could influence the fixation patterns and in the other direction, the eye movement pattern is tied to the scene representation in memory.

### 5.1. What Is Remembered of a Scene from a Fixation?

Fixation location provides a useful marker for what is attended in a scene and what can be processed with the highest acuity. It stands to reason that the memory for scenes would similarly be tied to fixation location. Although there have been claims that little to no memory exists of scenes [113,114], more recently the general consensus is that scene information can be retained reasonably well [115]. Thus, it is not a question of the visual representation’s existence, but the degree to which visual details are stored.

As highlighted earlier, the question of task is critical when examining eye movements. This question is just as critical when examining the interaction of eye movements and memory. What one is doing when fixating an object or part of a scene influences the quality and robustness of the memory retained. Some tasks encourage the participant to try explicitly to encode the information that is presented [116,117]. In other tasks, like visual search or aesthetic judgments, memory is retained incidentally [92,118,119]. Incidental encoding may be tested with an explicit memory test, such as a two alternative forced choice task (e.g., which of these hats did you see in a previously presented image? [111]), but encoding scene details was not the focus of the task. Finally, researchers also employ the relatively undefined task of “free viewing,” which may fall in between the explicit and incidental nature of encoding because the task itself does not provide an instruction of how to process the scene. Thus, participants are left to their own discretion as to how to process the image.

With regard to memory for scene representations, in most viewing tasks encoding is incidental in nature. Tasks such as navigating, searching, or judging the environment are far more common and scene representations are formed even without explicit instructions. For instance, Castelhano and Henderson [111] examined memory for objects in scenes that were acquired either incidentally or intentionally. They found that objects that were directly fixated were remembered better than chance regardless of the manner in which they were encoded. More recent studies have extended this finding to incidentally viewed parts of scenes and found that increased viewing of an area of the scene led to better memory performance [120]. Thus, studies have demonstrated that even without an intention to remember, fixated locations tended to be encoded.

The memory for objects that are encountered when viewing a scene are heavily influenced by both the fixation on the object and the task being performed. Draschkow and Võ [121] had participants perform a multistage search through the same environment where participants were told to find items to pack for a trip. They examined memory for objects that were relevant and those that were irrelevant to the task. They found that relevant objects to the packing task were remembered better than irrelevant objects. However, although greater fixation time on objects before they were targets did predict faster subsequent finding, the memory advantage of the relevant objects was not related to fixation time (see also [122,123]). This finding demonstrates a more complex interaction of the task and memory than simply one based on fixation time.

Another paradigm used to assess memory for scenes and target objects is the contextual cuing paradigm [124]. In this paradigm, after a target is found in the first block of trials, the viewer uses the knowledge of that scene to move the eyes efficiently to the previously found target in subsequent searches [125,126,127]. In this paradigm, rather than explicitly testing memory, representations of scenes and objects are shown through a decrease in the time to fixate a previously located target object. The speed up from one presentation to the next demonstrates that memory existed from the previous encounter that can be used when viewing the next presentation.

Although repeating the exact scene can be informative, it is also possible to demonstrate more generalized learning of specific contingencies across different scenes. Brockmole and Võ [128] found that people could learn and thus locate a target letter more quickly, when the target letter always appeared on a specific object in a scene (e.g., the letter appeared on a pillow across multiple bedroom exemplars). This learning and speeded fixation was even evident when a more general association was needed between a category of scenes and the target location. Clearly, memory is affected by the locations of fixations on the scene. However, this relationship is not an absolute in that simply knowing the amount of time a part of the scene is fixated is not a perfect predictor of memory. Instead, the relationship of fixation to memory is affected by the task performed and the role of individual object details in the task. Without considering both components, the relationship between fixation and visual memory can be opaque.

### 5.2. How Does Memory of the Scene Influence Current Fixations?

One would assume that having previously fixated an object in a scene would make it easier to find that object later, but that advantage may be limited [78,129]. Võ and Wolfe [119] had participants repeatedly search the same scene for different targets, while their eyes were tracked. Critically, because the same scene was being searched several times, the target object of the current search task had been a distractor on previous searches. Võ and Wolfe found that even though the current target had been viewed on previous trials, it did not significantly reduce the time to find that current target. Although the shift from distractor to target did not improve search performance, once the object had been found as a target, subsequent searches for that target object were facilitated. On the other hand, Hollingworth [130] attempted to replicate these results with more participants and found that there was indeed facilitation of having viewed distractor objects on subsequent searches for distractor-turned-target objects. Similar to the previous study [78], Hollingworth found that searching for a previously located target produced a much larger facilitation of search. These results indicate that the way in which an object is processed influences memory, but there is a general facilitation for previously processing an object, regardless of the type of processing.

Another means by which memory can influence eye movements is when an undetected change occurs in the scene. Memory for the previous object information within the scene can be demonstrated by longer subsequent fixations on a changed object compared to when the object has not changed [131,132,133]. For instance, Võ, Zwickel, and Schneider [131] showed that when an object changed location from one presentation to the next, gaze durations on the changed object were lengthened, even when that change was not explicitly detected. This implicit detection of a change via fixations demonstrates that eye movements can be more sensitive to some cognitive processes than explicit response.

It is clear that what is fixated can affect scene memory and vice versa. However, what about the pattern of eye movements themselves? Do people have to reenact the eye movements they made when learning the scene to remember the scene [56,134,135,136]? This scan path idea of Noton and Stark [134] argued that the memory for a scene would be improved if the eye movement pattern were repeated. However, subsequent research has indicated that in its strongest sense, the pattern of eye movements during retrieval does not have to match the pattern at encoding in order to recognize the scene. For example, Foulsham and Kingstone [56] found that there was no advantage of viewing one’s own pattern of eye movements on memory of a scene fixating compared to viewing someone else’s eye movement pattern. However, more recent studies have attempted to show that under limited circumstances, eye movements can be repeated between an initial viewing and a subsequent retrieval. Some of the stronger evidence for this claim comes from studies of “looking at nothing” studies. Johansson and Johansson [135] found that memory for objects was improved if people fixate where the object had previously been located (although the screen was blank at the time of retrieval) compared to when they fixate another location. This type of memory advantage extends to imagined scenes as well [136]. Thus, looking where something used to be appears to provide a boost to memory. In addition, it seems that there can be overlap of portions of the scan path from one viewing to the next [137,138]. Wynn et al. [137] found that fixations near the start and end of a search sequence were similar between viewings of the same scene in a change detection task. However, the fixations between the initial fixations on the scene and the final fixations on the scene did not match between the two views. Given the lack of similarity for most of the scan path and the heavy influence of the change detection task, these results provide limited support for scan path recapitulation as part of the retrieval process. In a similar vein, Bochynska and Laeng [138] compared a free viewing task (i.e., allowing participants to move their eyes) with a restricted viewing (i.e., participants could not move their eyes outside a central box) and found that memory for a sequence was better with free viewing compared to the restricted viewing. Although evidence that being able to move the eyes is better than not moving the eyes (see [139] for a similar finding with face stimuli), it is not strong evidence that eye gaze had to follow the same pattern to retrieve information. In general, although the execution of eye movements is important to remembering scenes, the recapitulation of the eye movements appears to add limited explanatory power.

Both the impact of memory on eye movements and the impact of eye movements on memory for the scenes clearly demonstrates that scene processing is an interactive process. In the first encounter with a scene, what is fixated and for how long influences what is stored in memory. At the other end, prior experience with a scene influences what is selected to be viewed and for how long. The fact that information can flow both directions indicates the strong connection between attention and memory. Although the idea is not new—William James pointed to the function of attention and the stream of consciousness in a similar way [140]—the ability to use eye movements as an observable method to measure this connection has greatly helped our understanding of the interaction.

As mentioned above, much of the research on scene perception has been limited to static scenes depicted on computer monitors in various formats. However, there has been significant progress in the research of scenes that more closely mirrors the experience of an observer when embedded in a scene. Next, we examine how representations differ as information changes over time and how eye movements differ when reflecting dynamic scene processing.

## 6. Dynamic Scenes and Eye Movements

A vast majority of the work examining eye movements and scene perception has used static images projected on a computer monitor. Limiting the scope of the research to static images makes sense when one considers the complexity of the stimuli. However, static images are a specific case of viewing compared to how the real world is processed. Dynamic environments can involve changes caused by the individual moving through or interacting with the environment [141,142,143,144] or by watching a dynamic scene unfold [145,146,147]. Although the studying of eye movements involved in the perception of dynamic scenes creates some technical challenges, the investigation of the how the eyes move in natural, dynamic scenes allows researchers to have a better understanding of scenes more generally.

Even though scenes can be dynamic in two ways (as mentioned above), most of the work in the last decade has focused on dynamic scenes that unfold over time before the viewer. The first question that arises in examining these dynamic scenes is the differences in eye movement patterns between static and dynamic scenes. Although they may contain similar information at different levels of analysis, dynamic scenes’ additional motion cues do alter the fixation patterns. The basic motion of dynamic scenes leads to larger differences between static and dynamic scenes than between different types of dynamic scenes [145]. Further, Mital et al. [146] examined the visual factors that predict eye movements while watching dynamic images. They found that motion within the dynamic scene was the most predictive of where the eyes would fixate. The differences between static and dynamic scenes even extend to the common laboratory findings, such as oculomotor capture, that occur regularly in static images, but they may be absent or altered in dynamic scenes [148].

Previous findings have also indicated that dynamic scenes’ motion cues lead to a higher degree of “attentional synchrony,” where people tend to look at the same location at the same time [38,145,149,150]. For instance, Dorr et al. [145] found that viewing professional movies resulted in fixation patterns that tend to cluster in the same regions of any individual shot. Similarly, Tseng et al. [38] attempted to disentangle the contribution of various viewing factors (e.g., saliency, photographer bias) on the production of the center bias in dynamic scene viewing and found that the tendency of points of high interest and salience to be located in the center of the image was the strongest contributor to the center bias. These common factors can be described as the “tyranny of film,” which has been shown to be a dominant factor in where people look in films [150]. These studies indicate that there are strong influences that encourage the viewer to cluster their fixations in a similar location, especially when the scene is changing over time. The question that remains is whether this is due to simple stimulus-based factors or is it a result of higher-level information being associated with motion. Interestingly, more naturally filmed scenes as well as static scenes are not processed with the same type of central focus that is seen with professionally filmed scenes [143].

However, viewing task seems to affect the similarity of fixation patterns between groups [151,152]. Smith and Mital [151] found that the highest level of cross-participant similarity in eye movements was associated with dynamic scenes during a free viewing task. This high degree of similarity indicates that fixation location is at least in part due to endogenous and task-related factors. Further, Foulsham and Kingstone [152] compared where people fixate while virtually walking around scenes on a computer screen. The comparison indicated that even when taking into account sequential information, eye movements on static scenes were not a better predictor of the location of real-world fixations than a model that simply relied on a central bias. Together, these results warrant caution when attempting to generalize eye movements from a static image to those in the real world [145].

The study of dynamic scenes is an increasingly promising area for the study of eye movements and scenes. Findings that eye movements in dynamic scenes do not correspond to those in static scenes leads to a need to develop new methodologies and possible new theoretical frameworks. Static scenes have provided a good starting point for researchers by providing more control over the stimuli. However, as eye movements change when the same information is presented dynamically, researchers need to consider how other factors could interact with the motion inherent in dynamic scenes. For instance, the task given the person or the effect of the immediately preceding history of an unfolding scene could both influence the fixation locations even with the motion of dynamic scenes. Moreover, of course, the interaction of the observer with the environment, such as when the observer is allowed to navigate in the real world, will require increasingly complex theoretical approaches. One tractable method of investigating dynamic scenes may be addressed through immersive environments (i.e., Virtual Reality) as they will allow researchers to have control over both types of dynamic scenes.

## 7. Conclusions

When Rayner and Pollatsek [5] reviewed the literature on eye movements and scene perception, they felt compelled to justify the use of eye movements as a way to study the cognitive processing of scenes. At that time, studies were demonstrating that scene representations could be formed with extremely brief presentations that were too short to allow for planning and executing eye movements [153]. As a result, researchers posited that measuring eye movements would not be particularly informative for scene perception because sufficient information was garnered without them. However, the explosion of eye movement research in scenes since that review, and especially in the last decade, clearly indicates that eye movement measures are indispensable as a tool for understanding scene processing.

We began this review by examining a number of influences that have motivated research in scene processing and eye movements. We reviewed research examining: (1) attention and eye movements; (2) where you look; (3) influence of task; (4) memory and scene representations; and (5) dynamic scenes. The research in each of these areas has advanced dramatically in the last decade and led to considerable improvement in our understanding of the visual processes involved in scene perception. However, what is not clear is where to go from here. Do we continue to drill down on each of these influences and possibly other factors that may have an effect or is there an alternative approach?

Although we have organized and discussed the five influences separately, we also made note of the different ways in which these influences could interact. Here, we hope to highlight that the future advancement of the field will be in examining these interactions directly. For example, although one can argue about the relative contributions of top-down and bottom-up processes, the way in which different sources of information (scene gist knowledge, expected layout, probability of location, and meaning, among others) contribute to processing may depend on other critical factors such as task, history/memory, and type of scene (embedded 3D or viewing 2D). This would require researchers to view these combinations not as a set of additive, independent contributions that are typically referred to as top-down effects, but rather begin to describe how certain critical factors and influences interact and lead to qualitatively different types of processing compared to other combinations. Rather than a list or a hierarchy, researchers need to move toward an understanding of how the landscape of “top-down” factors is shaped. For example, Awh et al. [51] posits that experience affects performance in a way that is different from previous homogeneous views of top-down influences. Defined as a form of selection history, they found an impact of the previous trials’ selections on the current trial on performance. The approach has begun the process of breaking down what we mean by top-down influences by including the unique contribution of experience.

The future of using eye movements to study scenes appears to be at the intersections of the areas we have described. By examining the intersectionality (processing that is unique to the specific combination) of these influences, we can begin to ask more complex questions. This argument is similar to that of Wertheimer (1923/1938) who, in comparing Gestalt Psychology to Structuralism [154], stated “I stand at the window and see a house, trees, sky. Theoretically I might say there were 327 brightnesses and nuances of colour. Do I have ‘327′? No. I have sky, house, and trees” ([155], p. 71). The recognition of the Gestalt Psychologists was that in attempting to break down the scene, the actual goal of understanding scenes was lost. A more holistic approach was needed. With respect to eye movements and scenes, a substantial amount of research has attempted to break down scenes into their components to understand the nature of viewing them. However, it will take looking beyond the components to the intersections, in order to gain a better understanding of the scene perception and eye movements.
